# COVIDium: a COVID-19 resource compendium

**DOI:** 10.1093/database/baab057

**Published:** 2021-09-29

**Authors:** Rohit Satyam, Malik Yousef, Sahar Qazi, Adil Manzoor Bhat, Khalid Raza

**Affiliations:** Department of Information Systems, Zefat Academic College, Jerusalem St 11, Safed, Zefat 1320611, Israel; Department of Computer Science, Jamia Millia Islamia, Maulana Mohammad Ali Jauhar Marg, Jamia Nagar, Okhla, New Delhi 110025, India; Department of Computer Science, Jamia Millia Islamia, Maulana Mohammad Ali Jauhar Marg, Jamia Nagar, Okhla, New Delhi 110025, India; Department of Computer Science, Jamia Millia Islamia, Maulana Mohammad Ali Jauhar Marg, Jamia Nagar, Okhla, New Delhi 110025, India

## Abstract

The severe acute respiratory syndrome coronavirus 2 that causes coronavirus disease 2019 (COVID-19) disrupted the normal functioning throughout the world since early 2020 and it continues to do so. Nonetheless, the global pandemic was taken up as a challenge by researchers across the globe to discover an effective cure, either in the form of a drug or vaccine. This resulted in an unprecedented surge of experimental and computational data and publications, which often translated their findings in the form of databases (DBs) and tools. Over 160 such DBs and more than 80 software tools were developed, which are uncharacterized, unannotated, deployed at different universal resource locators and are challenging to reach out through a normal web search. Besides, most of the DBs/tools are present on preprints and are either underutilized or unrecognized because of their inability to make it to top Google search hits. Henceforth, there was a need to crawl and characterize these DBs and create a compendium for easy referencing. The current article is one such concerted effort in this direction to create a COVID-19 resource compendium (COVIDium) that would facilitate the researchers to find suitable DBs and tools for their research studies. COVIDium tries to classify the DBs and tools into 11 broad categories for quick navigation. It also provides end-users some generic hit terms to filter the DB entries for quick access to the resources. Additionally, the DB provides Tracker Dashboard, Neuro Resources, references to COVID-19 datasets and protein–protein interactions. This compendium will be periodically updated to accommodate new resources.

**Database URL**: The COVIDium is accessible through http://kraza.in/covidium/

## Introduction

The coronavirus disease 2019 or COVID-19, caused by severe acute respiratory syndrome coronavirus 2 (SARS-CoV-2), has been declared as a global pandemic by the World Health Organization in early 2020 (1). The outbreak is currently going through multiple waves, costing the lives of more than 3.9 million people across the globe. The egregious pathogen belongs to the *Coronaviridae* family that affects both animal species and humans (2). Researchers have discerned that the most probable source of the SARS-CoV-2 are bats and its genome is said to have matching motifs such as the zinc motifs, DNA-binding domains and helix-loop-helix factors (3).

**Figure 1.**
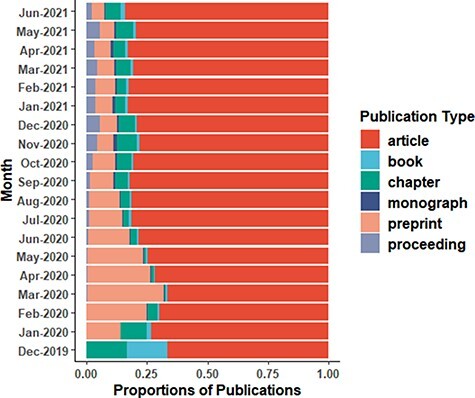
Proportions of publications related to COVID-19 and their month-wise distribution. The publications include articles, preprints, book chapters, proceedings, books and monographs. Data were obtained from the Dimensions DB, modified in R for visualization and plotted using ggplot2 with NPG color pallet of ggsci package (https://nanx.me/ggsci/).

The response of the scientific community to the pandemic is quite overwhelming and can be gauged by looking at the statistics of published literature (4). According to the Dimensions database (DB; https://reports.dimensions.ai/covid-19/), there are currently half a million publications associated with COVID-19, and it is poised to increase in the future (5). This can be visually represented in the form of month-wise proportions of various publication types as charted in Figure 1. Certainly, the flow of information in the COVID-19 era is intensifying and is driven by improved sequencing technologies, computational prediction algorithms and growing funding opportunities (6). As we speak, there are multitudes of novel aspects related to COVID-19 and the SARS-CoV-2 itself that are being unfurled (7, 8, 9, 10). These scientific findings are often communicated to the research community in the form of DBs and tool sets that can be easily accessed and/or deployed to test various pressing hypotheses. Since December 2020, many DBs have been published either in peer-reviewed journals or preprint servers or are shared on social media platforms such as Twitter for rapid dissemination of COVID-19-related information (11). These DBs are based on sequences (both nucleic acid and protein), structural data, epigenetic data, omics-based data (genetics, proteomics, transcriptomics, etc.), gene expression (microarray/next-generation sequencing), interactions, networks, disease-linked pathways and epidemiological data. Nonetheless, these DBs are highly scattered amidst the present corpus and remain underutilized because of their inability to appear in primary web searches. Efficient data exchange and management are central for developing holistic data-driven research and analysis of COVID-19 (12, 1). Ergo, it becomes imperative to accumulate all the COVID-19 DBs and tools and collate them in one place to make the data management and referencing easy for the researchers. Various repositories and compendiums have been prepared to disseminate pivotal knowledge about the rapidly evolving coronavirus (13, 14, 15, 12, 16, 17). The most popular compendiums include AccessClinicalData@NIAID which is a cloud-based data platform that helps in exchanging reports and datasets from National Institute of Allergy and Infectious Diseases (NIAID) COVID-19 and clinical trials for the research community. LitCovid (14) allows users to access numerous curated published peer-reviewed literature on COVID-19, whereas Nextstrain COVID-19 genetic epidemiology (https://github.com/nextstrain/ncov) is an open-source platform providing SARS-CoV-2 genome data for different analyses. Although informative, these resources are too descriptive and lack the logistics required by the users to quickly shortlist DBs and tools/ packages (such as programming language, user interface, specialized DBs, etc.). For instance, although European Virus Bioinformatics Center (EVBC) Virus Bioinformatics Tools (16) cover a generous breadth of COVID-19-related tools, their resource list is limited and minimal. Similarly, COVID19 Data Portal (12) enlists a minimal set of 33 DBs and atlases. The COVID-19 Resource Compendium (COVIDium) tries to provide an exhaustive list of resources, specialized DBs and a COVID-centric tool set and let users make informed choices about the resources most relevant to their query. It derives inspiration from the European Molecular Biology Laboratory’s European Bioinformatics Institute (EMBL-EBI) initiative of open data sharing via the COVID-19 Data Portal that systematically organizes a spectrum of COVID-19-related datasets (12).

The COVIDium currently enlists, categorizes and annotates 163 COVID-19-related DBs, 84 tools/packages developed for COVID-19 data analysis, 166 country/region-specific Tracker Dashboards, 5 neuro resources, 38 COVID-19 dataset resources and more than 18 000 protein–protein interactions (PPIs) collected from various interaction DBs enlisted in COVIDium. The motivation behind creating this compendium is to enable wider data sharing, exchange and analysis to help curb the ongoing outbreak of SARS-CoV-2. We believe that this compendium would serve as a unified resource for COVID-19 researchers, medical practitioners and pharmaceuticals. The DB will be periodically updated to accommodate new resources.

## Methodology

The methodology adopted to construct the COVIDium is described in the following subsections.

### Database source mining

A systematic literature review was carried out irrespective of the literature publication status to curate a list of DBs and tools/packages related to COVID-19 (18). Appropriate search terms were employed along with necessary AND and OR Boolean operators as and when required. The key terms included ‘COVID19’, ‘COVID-19’, ‘SARS’, ‘SARS-Cov-2’ and ‘Coronavirus’, combined with keywords ‘Database’, ‘Repository’, ‘Dashboard’ and ‘shinyapp’ for mining DBs and keywords ‘tools’ and ‘packages’ for COVID-19-related tools. The combinations used for querying DBs are precisely described in Annexure I. The DBs that were consulted for mining the literature were broadly divided into two levels: primary resources and additional resources. The primary literature DBs that were consulted include PubMed (https://pubmed.ncbi.nlm.nih.gov/), KDCovid (19, 20, 21, 22), LitCovid (7), Collabovid (https://www.collabovid.org/) and PubVenn (https://pubvenn.appspot.com/). The additional resources include bioRxiv, Embase, Google Scholar and Twitter. The abstracts obtained after querying the primary resources were reviewed manually by a team of three members. PubVenn helped us to improve the search by expanding the query terms with relevant Medical Subject Headings (MeSH) terms. The shiny dashboards were collected manually as well as from a recent work (23).

**Figure 2.**
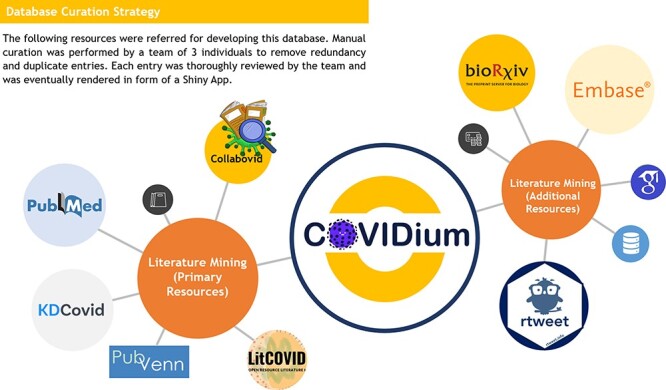
Database curation strategy adopted for the COVIDium.

The additional resources were also manually searched for the relevant literature. However, Twitter was crawled using ‘rtweet’ R package using search terms (#COVID-19 OR #SARS-CoV-2 OR #sars-cov-2 OR #Coronavirus 2 OR #NCOV OR #2019NCOV OR #COV AND #Database OR #shiny OR #dashboard) with a limit of 10 000 tweets. Retweets were not considered in our curation. The strategy of data source mining is charted in Figure 2, and a schematic of data extraction, processing and curation has been depicted in Supplementary Figure S1.

### Database classification

Based on the kind of data they present, the curated DBs were classified broadly into 11 categories that include Bibliographic DB, Genomic DB, Protein Sequence DB, Protein Structure DB, Drug DB, Interaction DB, Epidemiological DB, RNA DB, Pathway DB, Hybrid DB and Others. For instance, a DB hosting information about the variants, genomic sequences and phylogeny was wrapped in the umbrella term ‘Genomics DB’. Similarly, if the DB provided information about the PPI or drugs-related data they were categorized into ‘Interaction DB’ or ‘Drug DB’, respectively. DBs hosting a variety of datasets and were found to have multipurpose were categorized under ‘Hybrid DB’ [e.g. Outbreak.info and National Center for Biotechnology Information (NCBI) SARS-CoV-2 Resources]. Finally, the DBs that were hard to be categorized into the aforementioned categories were tagged ‘Others’. The tools and packages were also classified based on similar criteria. The standard definitions for each category are defined in Table 1.

**Table 1.** The standard definition of each category adopted in the COVIDium

Category abbreviation	Definition	Source
Bibliographic DB	A bibliographic DB refers to electronic records that provide a uniform description of a document, usually searchable by author name, title, subject header or keyword(s), or with citations and abstracts	(24)
Genomic DB	The Genomic DBs are online repositories of genomic variants, most of which are described for single or multiple genes or a specific population or ethnic group, intending to facilitate DNA-based diagnosis and correlating genomic variants with specific phenotypic patterns and clinical features. Gene expression DBs were also included	(25)
Protein Sequence DB	Protein sequences, peptides and other relevant information. These DBs store the features of proteins that are dictated by amino acid arrangement in protein sequences/peptides	(26)
Protein Structure DB	The DB contains information about the protein structures of SARS-CoV-2 that has been computed or determined experimentally. Structures that were modeled and simulated were also included	(27)
Drug DB	It contains information about drugs, inhibitors and chemical substances, as well as their targets	(28)
Interaction DB	Both drug–target interactions and PPIs are stored	(29)
Epidemiological DB	Epidemiological data refer to a variety of non-experimental findings, such as population exposure levels and health effect values discovered in samples	(30)
RNA DB	It contains information about RNA families and structured RNA elements	(31)
Pathway DB	Pathway DBs are a means of associating proteins with their functions and linking them into networks that characterize an organism’s reaction space	(32)
Hybrid DB	It considers a variety of genomic, proteomic and systems biology data such as variants information, interactions, sequences, peptides and literature altogether	This paper
Others	This category contains resources that could not be categorized into any of the aforementioned categories	This paper

### Annotation

The annotation enables more refined and sensitive index term searching within the DB. DB cross-references are followed and high-level textual annotation is added, describing the current state of biological knowledge about COVID-19. These data come from a variety of sources, notably from the primary literature. All the information and knowledge described in the novel scientific literature has been translated into entries in many different sections of the COVIDium DB, making it possible to make it one station of information on COVID-19 without having to manually review the literature on it. The annotation of each DB and tool was carried out by a team of three members and further cross-verified by the other members. The potential conflicts were resolved using majority voting rules. An effort has been made to make the communication of users with DBs well defined and transparent by providing links to the sources of different DBs present in COVIDium. Table 2 briefly describes the controlled vocabulary that is used in the COVIDium DB.

**Table 2.** Different annotations along with the specific hit terms and resources used in the COVIDium

Assigning categories	Hit terms/Index terms	Various resources and information portals used in COVIDium
*Genes and genome*	Genes, genomic sequences, similarity, alignment, annotation, interactive visualization, variation, haplotype, mutation, gene expression, replication, gene ontology, epidemiology, domain, motifs, single nucleotide polymorphism (SNP)	GISAID, GESS, Pangolin COVID-19 Lineage Assigner, VADR—Viral Annotation DefineR, SARS-CoV-2 alignment screen, idCOV, MCCS, UCSC SARS-CoV-2 genome browser, GENOME DETECTIVE VIRUS TOOL, 2019nCoVR, Phyloscanner, CoV2ID, GLEAMviz, COVID-19 data portal, NCBI nucleotide sequences, SARS-CoV-2 data hub, ViPR SARS-CoV-2, VirHostNet, WOLFRAM, VAPOR, Ensembl COVID-19 resource, VBRC genome analysis tools, nextstrain, covidex, Haplotype Explorer, COVID-Align, CorGAT, CoV-Seq, SARS-COV-2 genome browser, COVID-19 Viral Genome Analysis Pipeline, VirusSeeker, COVIDOUTCOME—Estimating COVID Severity Based on Mutations in the SARS-CoV-2 Genome, COVIDier, CovRadar, CoVPipe, SNPnexus COVID, METATRYP Standalone Software
*Proteins and proteomics*	Proteins, proteomics, sequence, structure, protein expression, PPIs, function, molecular docking, simulation, localization, mutations, alignment, protein cleavage site, protein domains, post-translational modifications	COVIDier, MCCS, COVID-Align, COVID-19 Docking Server, CoViProteins, coronavirus3D, neXtProt, VIGOR4, UniProt COVID-19, COVID-19 molecular structure and therapeutics hub, NetCorona Server, COVIDep, NCBI protein sequences, PROSITE, PubChem COVID-19 data, STRING COVID-19 host-interactome, COVID-19 Simulator, SWISS-MODEL, the human protein atlas, VBRC, ViPR SARS-CoV-2, Virus-CKB, PoSeiDon, Pfam
*Transcriptomics*	RNA-seq, annotation, analysis, RNA secondary structures, RNA motifs	RNACentral, Rfam COVID-19 resources, viralrecon, VIRify, COVID19Net, SAveRUNNER, VADR, poreCov
*Phylogenetics and evolutionary analysis*	Phylogeny, phylogenetic trees, reconstructions, evolution, ancestor, ancestral analysis	BEAST 2, Phylogeographic reconstruction, Hypothesis Testing using Phylogenies (HyPhy), COPASI, MapMyCorona, Nextstrain, covidex, pangolin COVID-19 lineage assigner, 2019nCoVR, Phyloscanner, SARS-CoV-2 alignment screen, 2019nCoVR, CoV-GLUE, coronavirus typing tool, phylomeDB coronavirus phylomes, SARS-CoV-2 analysis workflow, Haploflow, PoSeiDon
*Interactions*	Interactions, mapping, protein–drug interactions, PPIs, network interactions	SAveRUNNER, COVID-19 UniProtKB, CORDIT, CoVex, COVID-19 disease map, IntAct, COVID-19 KnetMiner, P-HIPSTer, STRING, CCSB Virhostome, VirHostNet
*Pathway*	Pathway, SARS-CoV-2 pathways, pathway figures, signaling pathways	WikiPathways, CoV-Hipathia, KEGG
*Drugs*	Drug designing, drug repurposing, drug development, antivirals	CoVex, P-HIPSTer, CORDITE, VirHostNet, chemical checker, CoViLigands, D3SIMILARITY, D3targets-2019-nCoV, DrugBank, MolAICal, canSAR, COVID-19 docking server, Virus-CKB, Open Access CAS COVID-19 Antiviral Candidate Compounds, COVID-Vaccine Neuro AE, Coronavirus Antiviral and Resistance DB, COVID-19 Neuro Databank/Biobank (NeuroCOVID), New Antiviral Drugs for Treatment of COVID-19, Antiviral Therapy
*Hybrid*	Data integration, biocuration, knowledge graphs, visualization, imaging, deep neural networks, machine learning, simulation, ontology	COVID-19 TestNorm, COVID-19 Simulator, Coronavirus simulator, COVID19Net, DGL-KE, METATRYP Standalone Software, VirHostNet
*Literature, neuroscience resources and clinical trials*	Literature, neuroscience, curation, publication, searching, sorting	PubMed, Carrot2, COVID-Neuro Resource, CoroNerve, The Neurology and Neuropsychiatry of COVID-19, F1000Research, LitCovid, DB, COVID-19 research explorer, medRxiv, ClinicalTrials.gov
*Epidemiology*	Risk assessment, epidemiology, forecasting, planning, SEIR model	COVIDSIM, COVIDStrategyCalculator, GLEAMviz, oxcovid19
*Others*	Pathophysiology, risk assessment, surveillance, planning, simulation	COVID-19 planning tools, COVIDStrategyCalculator, MCCSX, Secure IBD: COVID-19 Risk Calculator

### Interactome

To make our DB more useful, we tried to build the interactome of SARS-CoV-2 using DBs we collected in the ‘Interaction DB’ category. The interactions were obtained from Network Maps Database (33), The BioGRID COVID-19 Coronavirus Curation Project (https://thebiogrid.org/project/3/covid-19-coronavirus.html), IntAct/IMEx Coronavirus dataset (http://www.ndexbio.org/#/networkset/4c2268a1-a0f0-11ea-aaef-0ac135e8bacf) (34) and SIGNOR 2.0 DB (https://signor.uniroma2.it/) (35). In the Network Maps dataset, we considered only 332 high-confidence interactions (33). Since each DB uses its own preferred IDs (gene symbols, protein IDs and generic names), we tried to primarily make the IDs uniform before combining the interaction information. The UniProt IDs were converted to respective unique gene symbols using mapIds() function of AnnotationDbipackage of R (36). The annotations for protein to gene ID mapping were obtained from org.Hs.eg.db (v3.11.4). However, the gene symbols for the virus proteins were obtained (https://egonw.github.io/SARS-CoV-2-Queries/) and manually added. The interactions were finally merged and an adjacency list was produced to make an undirected graph.

### Implementation

The COVIDium is a Shiny App written entirely in R language that uses custom Cascading Style Sheets (CSS) formatting and is hosted on the shinyApp server. The DB will be updated periodically and will be actively maintained by the authors in the light of ongoing COVID-19 pandemic.

## Web interface

The COVIDium consists of six panels namely Databases, Tracker Dashboards, Neuro Resources, Tools/Packages, Datasets and Interactome.

### Databases panel

It classifies 163 COVID-19-related DBs and makes it easy for users to quickly filter the table based on key terms. The key terms are generalized to make it feasible for all groups of researchers to use DBs. The users can also download the filtered table should it be required. The entries in the ‘Databases’ column will redirect the user to the associated DB. For some DBs, the dataset was either genome obtained from Global Initiative on Sharing Avian Influenza Data (GISAID) that is not publicly sharable (37, 38), available upon request, or not available and therefore demarcated as ‘NA’.

### Tracker Dashboard panel

It enlists several country-specific trackers that help keep a track of the current mortality rate, live reporting of cases, etc. These dashboards also enable users to download updated datasets and perform epidemiological analysis and demarcate the severely hit regions at a country level. Most of these dashboards enable end user to download updated data in real time. Currently, this panel holds Country/Region-specific 166 Tracker Dashboards (23).

### Neuro Resources panel

It is an effort to enlist the DBs that registers information about the neurological implications of COVID-19. Broadly speaking, this panel is destined to host resources related to neurological and neuropsychiatric manifestations and complications of COVID-19 infections. This panel is currently populated with five entries and will be updated regularly as the field grows.

### Tools/Packages panel

It enlists the tools and packages that are specifically developed by researchers across the globe to analyze the COVID-19-related data or have been used in COVID-19 research. We refused to include the general-purpose tools in our compendium; however, this does not rule out their utility for COVID-19-related research.

### Dataset panel

It aims at enlisting the datasets/data archives we filtered from the literature or found on GitHub that could be used for integrative analysis or other secondary analysis.

### Interactome panel

It helps users visualize the PPI network of SARS-CoV-2 proteins and Human Proteome. We were able to harvest 18 252 unique interactions after combining data from various interaction DBs.

## Applications and utilities of the COVIDium

**Figure 3.**
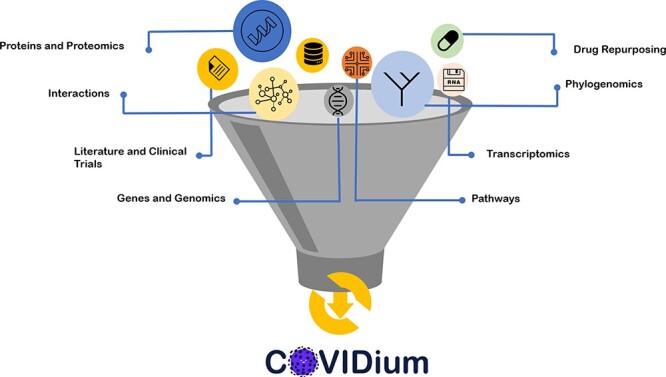
Types of DBs and tool sets enlisted in COVIDium.

The COVIDium would work as a single station for all COVID-19 DBs and tools, allowing researchers to search, filter, navigate and download SARS-CoV-2-related resources. This compendium currently stores 163 DB resources on COVID-19. Besides sequences, structures, interactions, disease, drug, epidemiological and bibliographic DBs, it also includes various machine-learning and artificial-intelligence-based software tools, analysis tools that aid in exploring the COVID-19 data in myriad ways. Our COVIDium encapsulates the powerful resources of literature biological and computational analysis tools that aid in understanding the COVID-19 and its newly identified seven variants of concerns. Figure 3 showcases various resources and information that have been accommodated in COVIDium for understanding COVID-19. Category-wise distribution of DBs in COVIDium representing the most abundant DB category is depicted in Supplementary Figure S2.

By default, for each search, the top 10 results are displayed on the main page, which can be changed to 25, 50 or even 100 results as defined by the user. Users can freely explore these different DBs and can also retrieve results by using hit terms such as mutations, tracker, genomes/genomic sequences, network/interactions/pathways, asymptomatic/symptomatic, evolution and immune signature. Apart from various DBs, we also have cross-referenced a few benchmark datasets from some reliable and renowned sources, for instance ‘KAGGLE’, ‘European COVID-19 Data portal’, ‘COVID-19 data portal’, etc.

Major applications of our COVIDium are being summarized as follows:

- ‘Current and up-to-date information’: The compendium allows users to fetch and retrieve the most recent set of resources recorded in the compendium, which are periodically checked, tracked and updated. Also, users can switch over to the literature category present in the Database panel and be redirected to the relevant Bibliographic Database in no time to retrieve articles published on different aspects of COVID-19.
- ‘Hit-term-based search strategy’: For an easy searching experience, COVIDium allows a hit-term-based searching strategy option for users to save their time and that they can have specific and precise search results. This is implemented in the form of Local and Global Search boxes and has been detailed out in the ‘Use Cases’ section. This way, users can retrieve the best possible hits for their queries.
- ‘SARS-CoV-2 datasets’: COVIDium points users to some recognized and verified benchmark datasets and data archives that can be used for different analyses. Since there are well-organized data archives already present such as COVID19 Data Portal (12) and COVID ARC (https://covid-arc.loni.usc.edu/), this panel is restricted to redirecting users to these well-organized archives.
- ‘Interactive network interactions and pathways’: Users can easily fetch and retrieve various network associations, interactions, pathways and specific SARS-CoV-2 interactors by simply selecting the ‘Interaction DB’ category from the drop-down menu available in the Database panel of COVIDium. These are combined interactions collated from different dedicated network and pathway DBs for COVID-19 and can be downloaded in the form of an adjacency list that can be visualized later in Cytoscape by the users as undirected graphs. Users can also subset the adjacency list before download for the desired COVID-19 genes.
- ‘Tracking COVID-19’: Surveillance, tracking and monitoring of COVID-19 have been a mess since it originated in 2020. Nevertheless, COVIDium provides epidemiological resources for our users to track and check the current status of the COVID-19 worldwide in real time in a country/region-specific manner. Some of these dashboards also provide raw data that can be downloaded to reuse to make predictive epidemiological models in a region-specific manner.
- ‘Fetching COVIDium data’: We provide an option ‘Download’ where interested users can download raw data or be redirected to the download page shall there be multiple files from COVIDium. The complete processed data tables (DTs) and networks can be downloaded from the GitHub repository.

### Use cases

Here we provide cases illustrating how COVIDium can quickly let you narrow down your search results.

#### Searching by keywords

Since COVIDium’s data are rendered in the form of DTs, users can use Local Search (Column Based Filtering) to filter DBs, tools and dashboards of interest, or use the Global Search option in the top right corner of each table to subset the entire table. For example, one can use Local Search to search the ‘Database’ DTs for the term ‘Drug’, which will subset the table based on the occurrence of the ‘Drug’ keyword while limiting the search to that column only (See Figure 4A). Partial matching using Global Search is also an alternative, as demonstrated in Figure 4B, where we employ ‘Pr’ to subset the DTs. This will show all the records (rows) having a word containing ‘Pr’ such as ‘Protocols’, ‘Protein’ or name of the DB itself such as ‘PROSPERO’. These keyword searches are case insensitive.

**Figu re 4.**
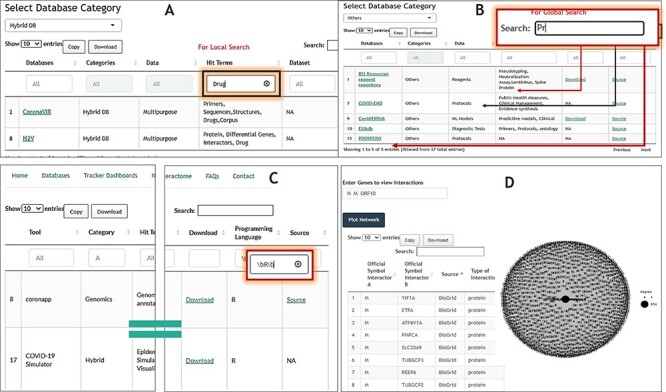
Schematic of COVIDium user interface highlighting features for searching and shortlisting using keywords (A and B) and regex (C).

#### Searching by regex

COVIDium searches are further made powerful for advanced users by enabling regex searches to subset the DTs. The regex might come handy when the user wishes to subset the entire DT such as ‘Tools/ Packages’ panel table using ‘pattern matching’. For instance, a user proficient in R might be interested in tools/packages written in R and therefore can subset the DTs locally using ‘Programming Language’ Local Search box and regex\bR\b, where ‘\b’ will treat R as a single word instead of a character. This will ensure that ‘Perl’-based tools do not surface in the results (See Figure 4C).

#### Protein–protein interactions

COVIDium also provides processed PPI data integrated from different resources using a uniform protein nomenclature. The interactions are rendered in the form of adjacency list, which can be copied or downloaded into .csv, xlsx or pdf format. The .csv file then can be loaded to Cytoscape to visualize the network.

### Limitations of COVIDium

COVIDium is a cross-referencing DB. It structures the existing DBs’ knowledge methodically and redirects the user to the relevant DB, data archives, tools, workflows, references, etc. instantly. However, it possesses some limitations too. COVIDium currently cannot perform Cross-Database queries. Cross-Database queries require collaboration and permissions to read data from the DB of interest. Since the number of DBs was high and we had limited resources with no funding, we restricted ourselves to cross-referencing presently. Besides, not all DBs have application programming interface and some DBs use GISAID data which cannot be shared publicly and require access requests (37, 38).

## Conclusion

The COVID-19-related research resulted in an unprecedented surge of experimental data and computational data, and publications translated their findings in the form of various DBs and tools. Here, we presented a COVIDium that was developed by crawling DBs, tools and trackers from various sources. Over 160 such DBs, more than 80 software tools, around 170 country-specific tracker dashboards and over 18 000 PPIs were crawled, characterized, annotated and stored in COVIDium for easy referencing.

In summary, COVIDium is a powerful resource that helps in bringing together published and unpublished DBs, tools, packages, datasets and dashboards to students, clinicians and researchers, in a curated and well-annotated form. Our compendium is unique as it compiles, for the first time, an exhaustive list of COVID-19-related resources in one place without the user requiring to perform a separate literature search for data, DB and tools identification. Such a resource can help users in quick decision-making and filtering pertinent resources for hypothesis generation and data analysis. With this resource, we hope to catalyze the pace of COVID-19 research and prevent ‘Reinvention of The wheel’ by informing the user about the existing DBs, pre-processed data, analysis pipelines and workflows, thereby saving them time from redundant efforts. The enlisted DBs in COVIDium will hopefully drive clinical and scientific implications in a positive direction. The COVIDium will be periodically updated and maintained by the team members to accommodate missing or newly developed resources.

## Supplementary Material

baab057_Supp

## Data Availability

Data are made freely available on GitHub: https://github.com/Rohit-Satyam/covidium and will be versioned to track changes.

## Annexure I

**Database search**: The primary results for COVID-19-related DBs were obtained from PubVenn to attain a visual understanding of the size of the data. Besides, PubVenn also facilitates extensive and accurate querying of PubMed by adding a series of additional MeSH terms. The DB was last queried on 1 August 2021. The following search queries were eventually used to retrieve the relevant literature from PubMed and other DBs:

**Query 1:**

"COVID-19"[All Fields] OR "COVID-19"[MeSH Terms] OR "COVID-19 Vaccines"[All Fields] OR "COVID-19 Vaccines"[MeSH Terms] "SARS-CoV-2"[All Fields] OR "sars-cov-2"[MeSH Terms] OR "Severe Acute Respiratory Syndrome Coronavirus 2"[All Fields] OR "NCOV"[All Fields] OR "2019 NCOV"[All Fields] OR (("coronavirus"[MeSH Terms] OR "coronavirus"[All Fields] OR "COV"[All Fields]))) AND ("Database"[Journal] OR "Database (Oxford)"[Journal] OR "database"[All Fields]).

**Query 2:**

("COVID-19"[All Fields] OR "COVID-19"[MeSH Terms] OR "COVID-19 Vaccines"[All Fields] OR "COVID-19 Vaccines"[MeSH Terms] "SARS-CoV-2"[All Fields] OR "sars-cov-2"[MeSH Terms] OR "Severe Acute Respiratory Syndrome Coronavirus 2"[All Fields] OR "NCOV"[All Fields] OR "2019 NCOV"[All Fields] OR (("coronavirus"[MeSH Terms] OR "coronavirus"[All Fields] OR "COV"[All Fields]))) AND Repository[All Fields].

**Query 3:**

("COVID-19"[All Fields] OR "COVID-19"[MeSH Terms] OR "COVID-19 Vaccines"[All Fields] OR "COVID-19 Vaccines"[MeSH Terms] "SARS-CoV-2"[All Fields] OR "sars-cov-2"[MeSH Terms] OR "Severe Acute Respiratory Syndrome Coronavirus 2"[All Fields] OR "NCOV"[All Fields] OR "2019 NCOV"[All Fields] OR (("coronavirus"[MeSH Terms] OR "coronavirus"[All Fields] OR "COV"[All Fields]))) AND Dashboard[All Fields].
