# Supplementary figures and images for: COVIDium: a COVID-19 resource compendium
Source: Database (Oxford). 2021 Sep 29;2021:baab057. doi: 10.1093/database/baab057 (PMC8500058; doi:10.1093/database/baab057)

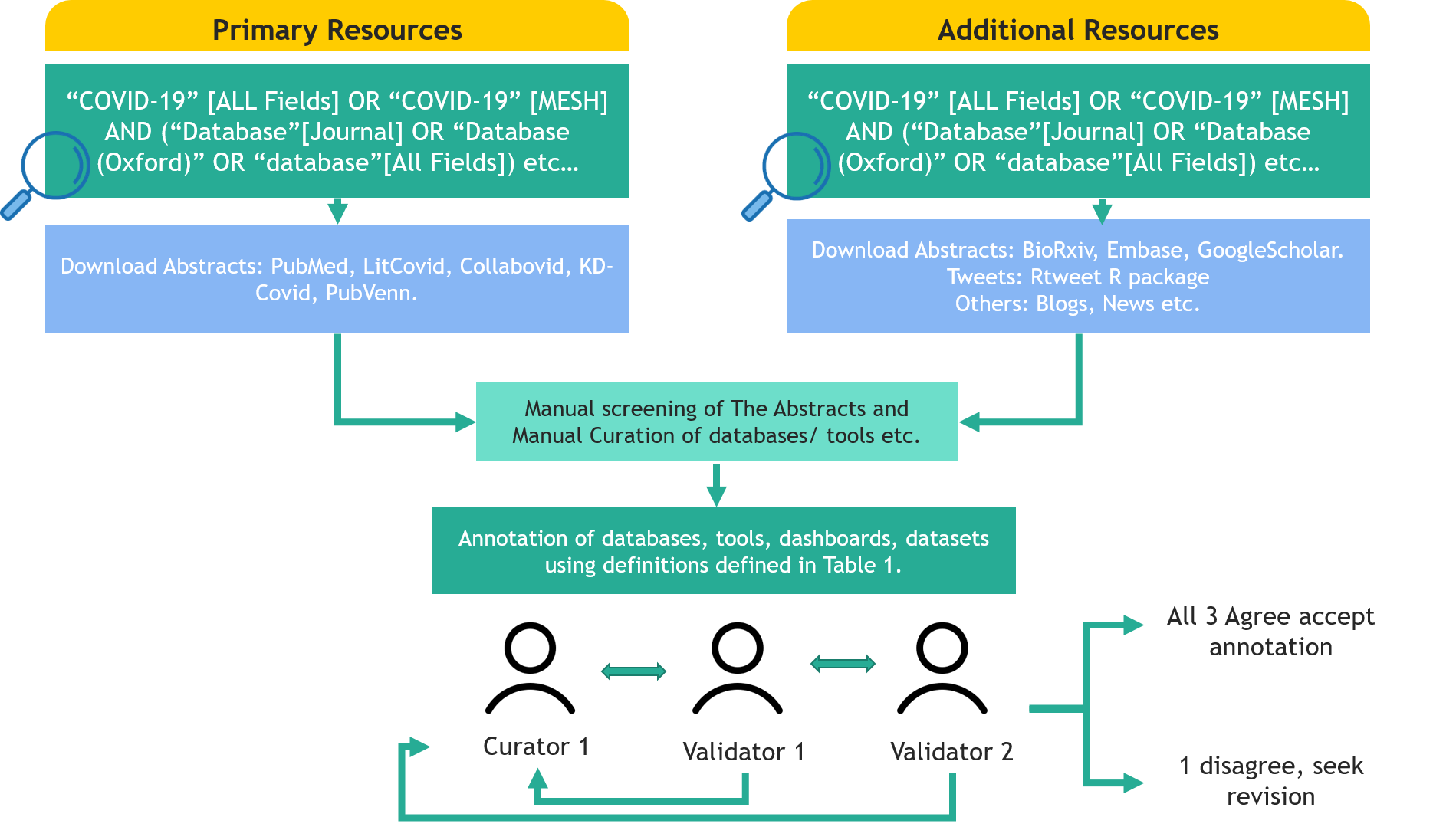

Supplement: baab057_Supp [file baab057_supp.zip › Supplementary Fig S1.png]

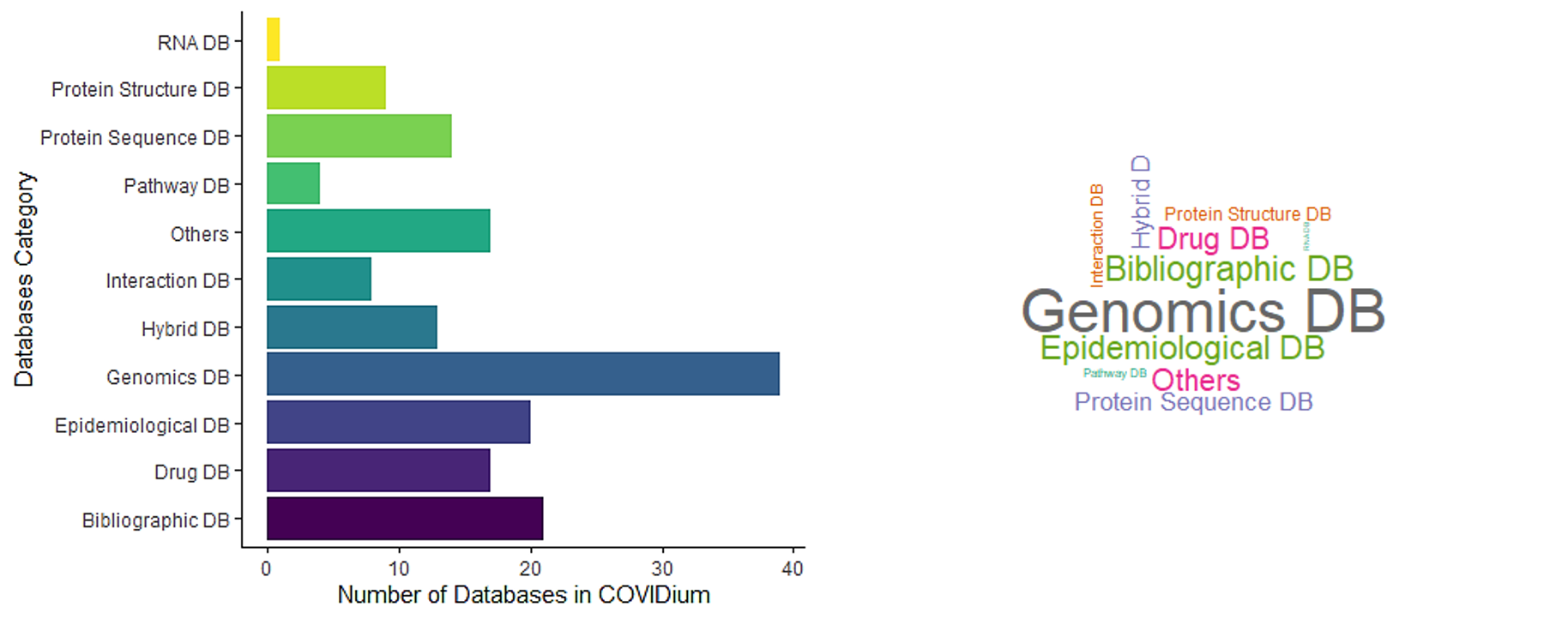

Supplement: baab057_Supp [file baab057_supp.zip › Supplementary Fig S2.png]
